# Chromosome conformation capture technologies as tools to detect structural variations and their repercussion in chromatin 3D configuration

**DOI:** 10.3389/fcell.2023.1219968

**Published:** 2023-06-29

**Authors:** Aura Stephenson-Gussinye, Mayra Furlan-Magaril

**Affiliations:** Molecular Genetics Department, Institute of Cellular Physiology, National Autonomous University of Mexico, Mexico City, Mexico

**Keywords:** chromatin, chromosome conformation capture (3C), structural variation (SV), chromatin architecture, topologically associated domains

## Abstract

3D genome organization regulates gene expression in different physiological and pathological contexts. Characterization of chromatin structure at different scales has provided information about how the genome organizes in the nuclear space, from chromosome territories, compartments of euchromatin and heterochromatin, topologically associated domains to punctual chromatin loops between genomic regulatory elements and gene promoters. In recent years, chromosome conformation capture technologies have also been used to characterize structural variations (SVs) *de novo* in pathological conditions. The study of SVs in cancer, has brought information about transcriptional misregulation that relates directly to the incidence and prognosis of the disease. For example, gene fusions have been discovered arising from chromosomal translocations that upregulate oncogenes expression, and other types of SVs have been described that alter large genomic regions encompassing many genes. However, studying SVs in 2D cannot capture all their regulatory implications in the genome. Recently, several bioinformatic tools have been developed to identify and classify SVs from chromosome conformation capture data and clarify how they impact chromatin structure in 3D, resulting in transcriptional misregulation. Here, we review recent literature concerning bioinformatic tools to characterize SVs from chromosome conformation capture technologies and exemplify their vast potential to rebuild the 3D landscape of genomes in cancer. The study of SVs from the 3D perspective can produce essential information about drivers, molecular targets, and disease evolution.

## Function of 3D chromatin architecture

The spatial organization of chromatin within the nucleus is an important layer of transcriptional regulation. Over the past decades, different levels at which chromatin is folded have been described, giving rise to different new scales of genomic regulatory landscapes (Bonev and Cavalli, 2016).

The study of genome topology at high resolution has been possible through the development and application of technologies derived from Chromosome Conformation Capture (3C) techniques, especially through Hi-C (Lieberman-Aiden et al., 2009). This technique measures genomic interactions by proximity ligation within the nucleus, which allows exploration of chromatin configuration in a specific cellular context. The Hi-C technique has allowed identification of different organization levels, such as A/B chromatin compartments, Topologically Associated Domains (TADs), and regulatory loops between distal elements in the genome (Lieberman-Aiden et al., 2009; Dixon et al., 2012; Rao et al., 2014).

The alteration of genome conformation at different scales has been reported in several pathologies. For example, it has been widely described that remodeling at the TAD level especially in the ability of these structures to isolate and therefore organize local interactions, gives place to alterations in the formation of the limbs during embryogenesis generating ectopic interactions between distal regulatory regions and developmental genes (Lupiáñez et al., 2015). This phenomenon has also been observed in pathologies like cancer, in which ectopic interactions between enhancers and oncogenes derive in the activation of oncogenic activity (Spielmann et al., 2018; Deng et al., 2022).

Also in cancer, a generalized phenomenon of DNA hypomethylation has been described (Nishiyama and Nakanishi, 2021) and recently, by studying the 3D structure of the genome in colon cancer, it has been observed that these hypomethylated blocks alter genome three-dimensional organization at the compartmental level, generating the movement of compartment B towards the center of the nucleus (Johnstone et al., 2020). Understanding the importance and complexity of 3D genome misfunction in different cancer types is still a growing field that could shed light on molecular mechanisms underlying the onset of cell transformation.

## 3C based technologies as tools to detect structural variations in altered genomes

In recent years, the Hi-C technique has been used not only to analyze the three-dimensional structuring of the genome, but also to identify chromosomal rearrangements. This approach is possible considering that in the data from Hi-C experiments, the contact probability between two loci decreases as a function to the genomic distance and that the likelihood of interaction between two regions within the same chromosome is greater than the one between regions in different chromosomes due to the organization of the genome in chromosome territories (Lieberman-Aiden et al., 2009).

The first application of these technologies in the identification of structural variations (SVs) was using Chromosome Conformation Capture on Chip (4C). Distinct interactome shapes related to sharp transitions in the data were observed and derived in the description of abnormal interactions as SVs. This technique allowed the identification of several genomic rearrangements present in the T cell–derived acute lymphocytic leukemia (T-ALL) cell line HSB-2. By analyzing the changes in the interaction patterns of the targeted regions the authors could observe the reciprocal translocation between chromosome 1 and 7 t (1;7) (p35;q35) and an inversion in chromosome 7 inv (7) (p15;q35) both previously reported in these cell line. 4C could also identify unbalanced translocations and new SVs targeting a locus frequently involved in chromosomal rearrangements (Simonis et al., 2009).

Years later, Hi-C was used for this purpose on HeLa cells. The visual observation of “off-diagonal patches of strong linkage with asymmetric decay” (Burton et al., 2013) in the Hi-C whole genome matrix were described as possible SVs. Then these patches were compared with already reported chromosomal rearrangements in these cells, and indeed the atypical interaction patterns corresponded to previously described SV markers. Additionally, this technique allowed them to describe new SVs that were not reported previously. The use of Hi-C to detect SVs was also confirmed using lymphoblastoid cell lines with known chromosomal rearrangements, in which the interaction matrices allowed the visual identification of balanced and unbalanced inter-chromosomal translocations (Burton et al., 2013; Harewood et al., 2017).

Over the last decade, several bioinformatics tools have been developed to use proximity ligation data to identify and characterize SVs such as deletions, inversions, duplications, and translocations of genetic material. Detecting SVs through 3C-based technologies has advantages over other strategies such as whole genome sequencing (WGS), genomic hybridization, and karyotyping. For instance, the capacity for detecting copy number neutral events is a feature that coverage-based methods cannot recall. Also, SVs that are difficult to identify as the ones located at repetitive regions can be recognized by Hi-C by observing the contact patterns of the regions surrounding the event. In addition, the sequencing depth needed to identify SVs visually in Hi-C data is lower compared to WGS experiments. The possible limitations of using this kind of data for detecting SVs are that most Hi-C experiments won´t identify small rearrangements due to the restriction fragments size and matrices resolution depending on the sequencing depth (Burton et al., 2013; Harewood et al., 2017; Chakraborty and Ay, 2018; Kim et al., 2022).

Another feature relevant to the use of 3C technologies in the identification of SVs relies on the capability of these techniques to unravel more complex mechanisms behind previously identified SVs. Hi-C was used in patients with various developmental diseases to characterize SVs using different tissues. It was found that a previously reported altered zone in chromosome 17 encompassing the *SOX9* gene, had tridimensional repercussions derived from SVs. These could explain some of the transcriptional dysregulation effects related to the diseases that could not be addressed through previous SVs identification methods (Melo et al., 2020).

Although some limitations are present, proximity ligation data could be an effective way to capture SVs at low resolution with low sequencing costs in a clinical framework. Inter-chromosomal translocations have been observed in HeLa cell line using Hi-C experiments with around 154 million valid pairs (Burton et al., 2013) and in glioblastoma samples using low sequenced Hi-C with less than 20 million valid pairs (Harewood et al., 2017). Also in lymphoblastoid cell lines carrying previously detected SVs, by using sequential reduction of Hi-C sequenced reads from 100 M down to 1 M it was found that with 50 M reads an intra-chromosomal tandem duplication was visible, and even with 1 M pair reads, a reciprocal translocation could be identified (Melo et al., 2020). It has been reported that even though the identification of differential loops is closely related to the sequencing depth, the differences between the number of SVs found in experiments of 1 and 3 billion reads is not highly different (Lee et al., 2022). This means that these techniques could be used in conjunction with other methods to further characterize rearranged genomes such as heritable germinal SVs, or as a diagnostic tool in personalized medicine.

The first bioinformatic tools developed to detect SVs and copy number variations (CNV) from Hi-C were published in 2018. HiCnv and HiCtrans pipelines were designed to find CNV and chromosomal translocations, respectively. HiCnv can predict large-scale CNV larger than 1Mb, and HiCtrans can recognize inter-chromosomal translocations from a 10 kb resolution Hi-C matrix and report their breakpoint at restriction site or lower resolution (Chakraborty and Ay, 2018).

**FIGURE 1:**
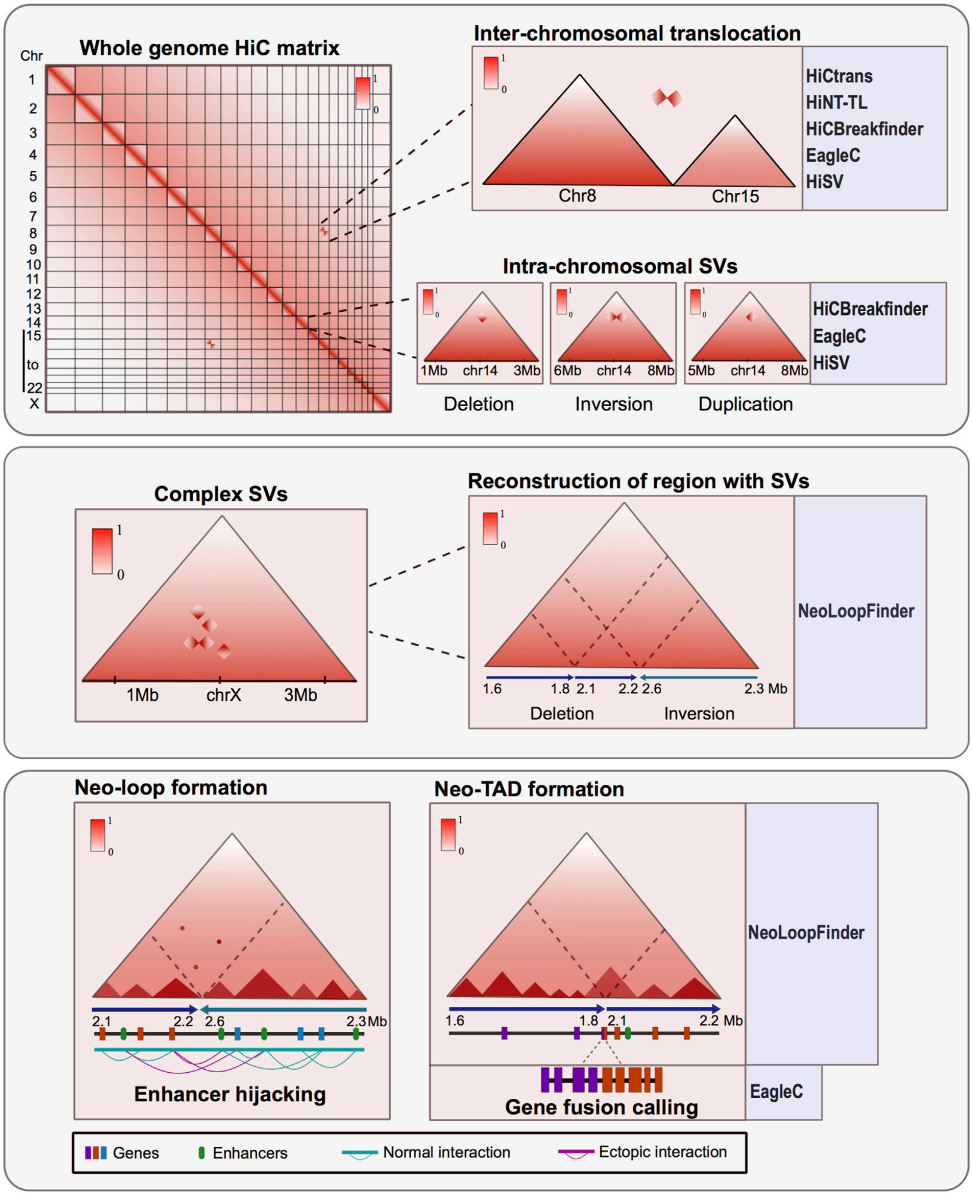
Hi-C is a powerful tool for characterizing structural variants (SVs) in altered genomes. Upper panel: Matrix derived from Hi-C experiments from rearranged genomes shows atypical interactome patterns that can represent inter-chromosomal translocations or intra-chromosomal SV. These alterations can be observed in the matrices and also can be identified and annotated by various bioinformatic tools. Middle panel: Hi-C data can identify complex structural variants like chromothripsis and permit the reconstruction of the region affected by an SV using the interaction patterns. Lower panel: SV can result in the formation of new topologically associated domains and interaction loops between the altered regions. The consequences of these rearrangements could trigger gene fusions, ectopic interaction and enhancer hijacking events. There are bioinformatic tools developed to assess the tridimensional consequences of SV using Hi-C data.

Subsequently, various bioinformatic approaches were developed to identify SVs at inter and intra-chromosome resolution (Figure 1). Among these pipelines HiCnv, HiNT, especially the HiNT-CNV algorithm, and NeoLoopFinder are able to find CNV using Hi-C data; on the other hand, HiCtrans, HiCBreakfinder, HiNT-TL, EagleC, and HiSV (Hi-C for Structural Variation), can assess SVs using the interactome patterns as a way to validate their findings. HiCBreakfinder, EagleC, and HiSV can report the presence of SVs within chromosomes. The distinct interaction patterns seen in the matrices can be translated to the type of chromosomal rearrangement (Chakraborty and Ay, 2018; Dixon et al., 2018; Wang et al., 2020; Wang et al., 2021; Wang et al., 2022; Li et al., 2023) (Figure 1).

All these tools apply different metrics to identify SVs, for which the visual inspection of the maps and other methods such as the recapitulation of breakpoints using PCR techniques, have helped to discriminate false positives and corroborate the results reported by the programs (Adeel et al., 2022).

Also, there are other tools to further characterize the impact of the SVs in genomic regulation and function. For instance, in NeoLoopFinder there are features that allow identifying neo-TADs formed as a result of SVs and events of enhancer hijacking when other epigenetic data such as enrichment of histone modifications is available (Wang et al., 2021) (Figure 1). Also, EagleC allows to recall gene fusions resulting from SVs (Wang et al., 2022) (Figure 1), and SVInterpreter, a website application, helps to predict the phenotypic outcome and the possible clinical implications of several types of SVs (Fino et al., 2021).

Another advantage arising from the analysis of proximity-ligation data is the characterization of complex rearrangements like chromothripsis, frequently found in cancer. These complex SVs can be visualized in the Hi-C matrices as zones with many ectopic interactions forming distinctive patterns. Assessing the regulatory consequences of these complex SVs represents a challenge. However, by using Hi-C, it is possible to manually reconstruct the altered chromosomes and determine the tridimensional consequences of such SVs (Cortés-Ciriano et al., 2020; Schöpflin et al., 2022; Sidiropoulos et al., 2022). Other bioinformatic tools have been developed to reconstruct SVs in a tridimensional context. These tools allow the identification and exploration of ectopic TADs and neo-loops formed mainly in cancerous genomes (Wang et al., 2021) (Figure 1).

In addition to the bioinformatic pipelines, new experimental protocols have been developed that allow using Hi-C in samples from patients with low cell input and derived from solid biopsies, the most common type of sample in adult cancer diagnosis. The protocols adapted to perform efficiently in low cell numbers have a very high and important potential to be applied to call SVs in the clinical context (Díaz et al., 2018; Animesh et al., 2021).

The tools to analyze Hi-C data to detect SVs and CNVs have opened up a broad and exciting field for the characterization of chromosomal rearrangements from the 3D perspective and characterize transcriptionally altered regulatory landscapes.

## Characterization of SVs in cancer using 3C techniques

Cancer is a complex and heterogeneous pathology that currently represents one of the major health challenges world wide both from a clinical and a research perspective. There are various layers of dysregulation during cellular transformation leading to cancer, and the characterization and understanding of each are vital in the search for new therapeutic targets and therapies that can help improve the prognosis and quality of life of the diagnosed patients.

SVs have been studied for a long time in different types of cancer both as drivers and as consequences of the genomic instability characteristic of cancer cells. It has been described that different SVs can predispose specific populations to suffer from certain types of cancer (Bonassi et al., 2004). Also, there are a lot of events described in which SVs are related to transcriptional misregulation. For example, in breast cancer, some SVs generate gene fusions (Banerji et al., 2012) and in myeloma, some SVs alter the *cis*-regulatory landscape of genes disrupting their expression and worsening the prognosis of the patients (Walker et al., 2014). However, many of the consequences of SVs have been studied only from a two-dimensional perspective then missing the full disruption of the regulatory landscape. For instance, in pancreatic cancer cell lines, deletions in the *CDKN2A* and *SMAD4* genes, both of which are common in pancreatic cancer tumors, have in addition to a direct consequence on the coding gene, a three-dimensional consequence altering the contact domains in the area (Du et al., 2022).

SVs in cancerous genomes can promote the formation of ectopic interactions, new TADs, and enhancer hijacking events, all of which can be numerous and heterogeneous depending on the cancer type. Nevertheless, not all tridimensional alterations derived from SVs have large transcriptional consequences (Ghavi-Helm et al., 2019; Akdemir et al., 2020). Thus, it is essential to delve further into the characterization of specific chromosomal variations to understand the relationship between SVs, the tridimensional landscape and gene expression misregulation in a personalized manner.

SVs leading to TAD fusions have been reported among diverse cancer cell lines and tumor samples involving recurrent loci of oncogenes like *MYC, TERT,* and *CCND1*. Specifically, the structural landscape of *MYC* was found to be altered in breast, osteosarcoma, neuroblastoma, lymphoma and pancreatic cancers. Transcriptomic data from tumors showed that several of the rearrangements were related with higher expression of the oncogene, even when the tumors did not have gains in locus copy number. Nevertheless, the transcriptional consequences of the SV in cancerous genomes are highly heterogeneous. TAD fusions are commonly involved in relocation of genes and *cis* regulatory elements, however, not all of them result in an alteration of transcription. Xu Z. et al. (2022) recently demonstrated using CRISPR-cas9 engineering, that genomic alteration of the *MYC* locus inducing an SV is not enough to disrupt its expression *per se*, but it is the presence, number, and strength of the enhancers included in the translocation what determines the transcriptional consequence of the rearrangement.

SVs can have different consequences in the same pathological process. For example in samples from ependymomal tumors, chromosomal rearrangements generated the dysregulation of several genes through different mechanisms including gene fusions related to SVs, ectopic gene-enhancer interactions, and a neo-TAD formation possibly related to disease relapse (Okonechnikov et al., 2023).

Taken together, these results highlight the importance of expanding our knowledge in the regulation pathways altered by the presence of SV from the genetic, epigenetic and topological perspectives to resolve the heterogeneous molecular nature of tumorigenic cells.

In T-lineage acute lymphoblastic leukemia various oncogenes are overexpressed both in patients’ samples and leukemia cell lines. One of the frequent mechanisms leading to gene expression misregulation in this disease is the alteration of insulated tridimensional neighborhoods. Deletion of CTCF DNA binding sites at the anchor of an insulated neighborhood, without altering the gene loci, resulted in the overexpression of well-characterized oncogenes like *TAL1* and *LMO2* (Hnisz et al., 2016).

In another study, 14 T-lineage acute lymphoblastic leukemia samples were analyzed using Hi-C. 46 translocations were identified, 34 of which were newly discovered and related to the formation of 44 neo-loops. The ectopic loops formed correlate with the clinical gene profile classification of the non-early T-cell precursor of acute lymphoblastic leukemia subtypes, suggesting that the tridimensional consequence of genome translocations could be a potential cause of the altered gene expression profiles previously described in this pathology. It was also reported that the over-expression of the *HOXA* cluster related to poorer prognosis in this type of cancer, could be associated with translocation-mediated neo-loop formations between genes in the *HOXA* cluster and highly active enhancers in the region (Yang et al., 2021).

**FIGURE 2:**
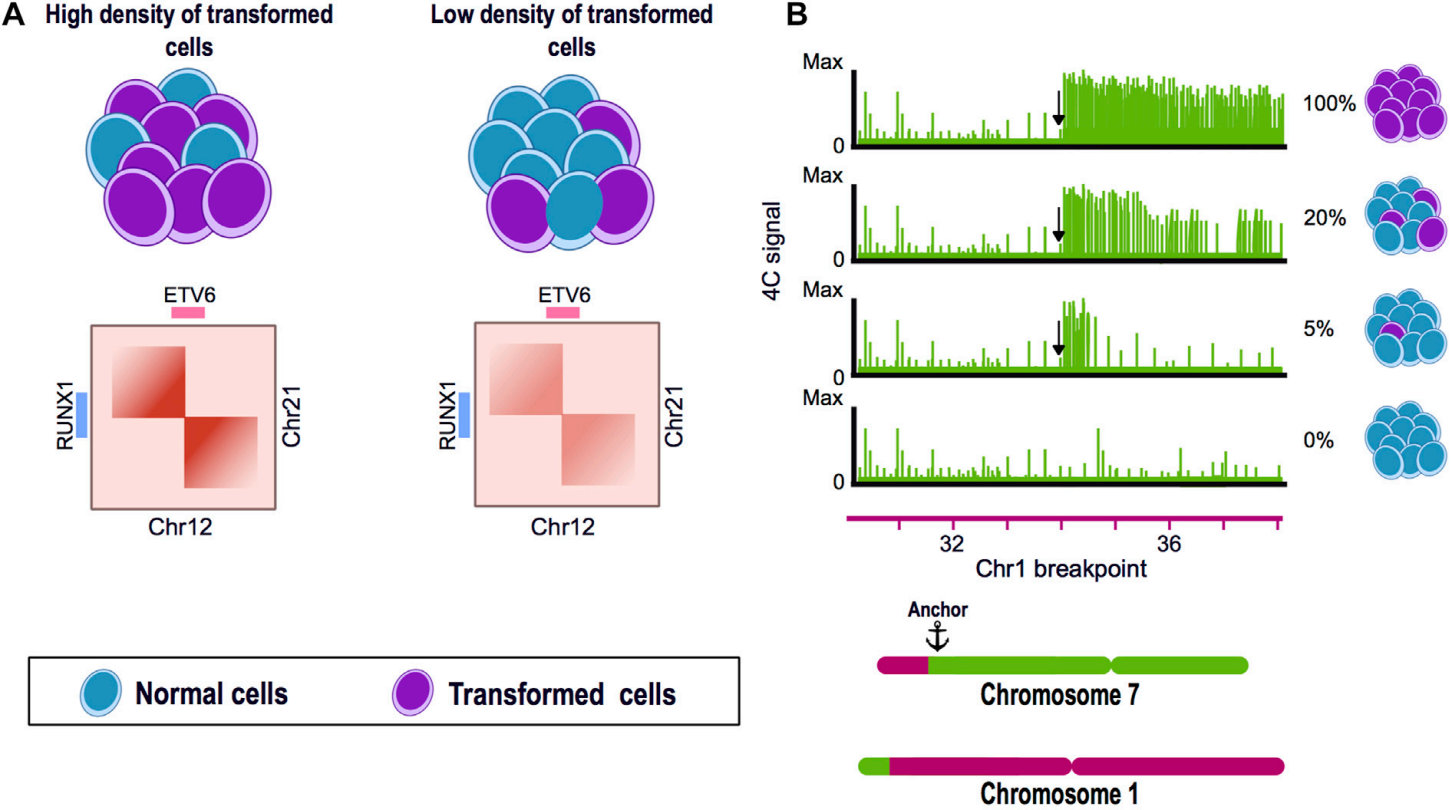
Chromosome conformation capture techniques as tools for the identification of structural variants. **(A)** Hi-C experiments can identify translocations present in a low percentage of the cells. Translocation of the chromosome 12 and 21 is a frequent genome alteration found in B-cell acute lymphoblastic leukemia (B-ALL) patients and results in the fusion of *ETV6-RUNX1* genes. Using a blood and bone marrow sample from a pediatric patient with B-ALL, a Hi-C experiment could identify the balanced translocation present in both samples and also allowed to visually assess the breakpoint of the SV. **(B)** 4C can identify genomic rearrangements in a very sensitive manner. A mixture of HSB-2 cell line harboring a known translocation t(1;7) and K562 cell line was analyzed by 4C using the region next to the breakpoint in chromosome 7 as anchor. The percentage of altered cells was diluted until 5% of the mixture presented the translocation. 4C data showed the presence of the SV even when only represented by a low percentage of the genomes in the mix.

Another study using samples of pediatric B-cell acute lymphoblastic leukemia found that shallow sequenced Hi-C experiments could recapitulate the SVs previously identified as drivers of this disease, such as the *ETV6-RUNX1* translocation found between chromosome 12 and 21. Using Hi-C data derived from blood samples, the authors efficiently identified SVs present in the patient´s bone marrow. These findings suggest that shallow Hi-C experiments are able to identify clinically significant SVs in samples with a low representation of altered cells and, thus, could improve the identification of SVs with a lesser invasive sampling method for the patients (Mallard et al., 2022) (Figure 2A).

In myeloid leukemia, the presence of SV generates several enhancer-hijacking events forming new chromatin loops between different enhancers and genes. Some of these genes, like *HSF4, MYC,* and *CBL,* present an upregulation pattern possibly derived from the incorrect activation by the hijacked enhancers. Another consequence of the tridimensional restructuration was “silencer hijacking”; 5.7% of the neo-loops formed were new interactions between genes and transcriptional silencers, for example; for *JAK1* and *KMT2C* genes that are both downregulated in the tumors (Xu J. et al., 2022).

Additional recent examples of the use of Hi-C data to characterize SVs are found in the analysis of cervical and bladder cancer, among others (Adeel et al., 2021; Iyyanki et al., 2021; Liu et al., 2023).

Together all this evidence shows that characterization of SVs from a 3D genomic perspective can add important information about potential drivers and regulatory consequences of chromosomal aberrations that need to be considered in a pathology with great molecular heterogeneity.

## Remarks and perspective

Understanding SVs from a 3D perspective is necessary to understand the potential causes and consequences that these can have in pathological contexts. The use of Hi-C and its derivatives has brought new understanding about how the genome folds and how this influences the formation of SVs. For example, it is known that SVs form preferentially between zones of the same compartment or similar replication timing (Sidiropoulos et al., 2022) which suggests that in specific genomic scenarios such as in the loss of DNA-repairing pathways, the gradual formation of SVs is influenced by the physical closeness and interactions between genomic regions. Additionally, chromosome conformation capture technologies have now been extendedly used to characterize SVs *de novo.*

Using Hi-C as a way to assess SVs has its limitations as it cannot find punctual mutations or small chromosomal variants. Nevertheless, it is possible to complement the analysis by using other technologies as WGS and leverage the capabilities of both techniques to achieve a complete characterization of the rearranged genomes (Li et al., 2019; Akdemir et al., 2020).

Characterizing the altered layers of misregulation in cancer is one of the most challenging endeavors in the field. Understanding the alteration of the 3D genome organization might resolve new and important facts about how this complex pathology behaves in patients.

Personalized medicine has become particularly important in heterogeneous pathologies like cancer and it is necessary to establish efficient and affordable methods to characterize tumor-specific genomic signatures. Tumor samples and biopsies are commonly very heterogeneous having a variable proportion of rearranged and normal somatic cells. In this matter, by using 4C technology, the SVs present in a mix of rearranged and normal genomes, could be efficiently detected having 5% and 95% of rearranged and normal cells respectively (Figure 2B). Even though the precise proportion of genomically abnormal cells in a heterogeneous mix needed for Hi-C to efficiently detect SVs has not been established, its potential has been demonstrated in leukemia patients, in order to detect chromosomal rearrangements from blood samples instead of bone marrow (Figure 2A). It is now necessary to assess in detail the sensitivity of Hi-C in the identification of SV, the amount of altered cells and the sequence depth needed in a clinical setup (Harewood et al., 2017; Animesh et al., 2021; Mallard et al., 2022).

Finding SVs through Hi-C has proven to be efficient and cost-effective and could be one of the most promising strategies to characterize, diagnose and propose better treatments in a personalized manner.
